# Updates in Drug Treatment of Severe Hypertriglyceridemia

**DOI:** 10.1007/s11883-023-01140-z

**Published:** 2023-08-29

**Authors:** Ioanna Gouni-Berthold, Jonas Schwarz, Heiner K. Berthold

**Affiliations:** 1 Center for Endocrinology, Diabetes and Preventive Medicine, University of Cologne, Faculty of Medicine and University Hospital, Kerpener Str. 6, 50937 Cologne, Germany; 2 Department of Internal Medicine and Geriatrics, Bethel Clinic (EvKB) and Medical School EWL, University of Bielefeld, Bielefeld, Germany

**Keywords:** Triglycerides, Hypertriglyceridemia-induced acute pancreatitis, Volanesorsen, Olezarsen, ARO-APOC3, ARO-ANG3

## Abstract

**Purpose of Review:**

To provide an insight into the new pharmacological options for the treatment of severe hypertriglyceridemia (sHTG).

**Recent Findings:**

sHTG is difficult to treat. The majority of the traditional pharmacological agents available have limited success in both robustly decreasing triglyceride levels and/or in reducing the incidence of acute pancreatitis (AP), the most severe complication of sHTG. Therapeutic options with novel mechanisms of action have been developed, such as antisense oligonucleotides (ASO) and small interfering RNA (siRNA) targeting *APOC3* and *ANGPTL3*. The review discusses also 2 abandoned drugs for sHTG treatment, evinacumab and vupanorsen.

**Summary:**

The ASO targeting *APOC3*, volanesorsen, is approved for use in patients with familial chylomicronemia syndrome (FCS) in Europe. Olezarsen, an N-acetylgalactosamine (GalNAc)-conjugated ASO with the same target, seems to have a better safety and efficacy profile. siRNA targeting *APOC3* and *ANGPTL3*, namely ARO-APOC3 and ARO-ANG3, are also promising for the treatment of sHTG. However, the ultimate clinical goal of any sHTG treatment, the decrease in the risk of AP, has not been definitively achieved till now by any pharmacotherapy, either approved or in development.

## Introduction

Severe hypertriglyceridemia (sHTG), defined as triglyceride (TG) levels of ≥ 880 mg/dl [1•] or in the American literature as TG ≥ 500 mg/dl [2] is an established risk factor for acute pancreatitis (AP). While the incidence of sHTG is 1:600 people, sHTG caused by the rare monogenic disorder familial chylomicronemia syndrome (FCS) is 1:100,000 to 1:1,000,000 [1•]. Multifactorial chylomicronemia (MCM), or multifactorial chylomicronemia syndrome (MCS), is by far the most common cause of sHTG. It is a polygenic disorder resulting from the combination of genetic variants and other factors negatively affecting TG metabolism such as a diet rich in fats and simple carbohydrates, obesity, alcohol intake, and poorly controlled diabetes [3]. For the purpose of simplicity, the terms MCM, MCS and sHTG will be used interchangeably in this review.

Diet remains the mainstay of treatment of any form of primary HTG, however, it is often not sufficient to bring patients to TG levels of < 500 mg/dl, the generally accepted threshold for the prevention of AP [4]. The traditional pharmacologic therapies for HTG such as statins, fibrates, niacin and omega-3 fatty acids, may offer some extra percentages of TG-lowering but are often insufficient to adequately reduce TG concentrations. In addition, no convincing data are available that these drugs prevent AP. Moreover, they require a functional lipolytic pathway so they may be of help in the “common” forms of sHTG but have minimal effects in FCS where this pathway is absent.

Statins, HMG-CoA reductase inhibitors, decrease TG levels by 10–20%, most likely via suppression of production and increased uptake of TG-rich lipoproteins [5]. Fibrates, peroxisome proliferator-activated receptor (PPAR) activators, can reduce TG levels by up to 50% in non-FCS patients with HTG and therefore have some meaningful role in the treatment of sHTG [6]. Niacin, with a still uncertain mechanism of action, can lower TG by 5–35% [7], but has been withdrawn from many markets due to its consistent failure to show any atherosclerotic cardiovascular disease (ASCVD) reduction in clinical trials. Omega-3 fatty acids, specifically eicosapentaenoic acid (EPA) can decrease TG levels by ~ 20% [8]. However, in some countries, like Germany, they are not reimbursed by statutory health insurance, limiting their availability. It is therefore clear that new pharmacologic agents offering a robust TG decrease are needed.

The goal of this review is to provide an insight into the new pharmacological treatment options for sHTG.

## Apolipoprotein C-III (apo-CIII) Inhibition

Apolipoprotein C-III (apo-CIII) is a small 79-amino acid glycoprotein encoded by the *APOC3* gene on chromosome 11q23.2. Apo-CIII increases plasma TG levels through various mechanisms such as reducing lipoprotein lipase (LPL) activity, the enzyme responsible for hydrolyzing TG, by inhibiting the removal of TG from the circulation and by promoting hepatic VLDL secretion into the bloodstream [9]. Moreover, it has been shown that loss-of-function (LOF) mutations in *APOC3* are associated with low levels of TG and a reduced risk of ischemic cardiovascular disease [10, 11]. Therefore, it was intuitive trying to inhibit *APOC3* in order to decrease circulating apo-CIII concentrations and decrease TG levels.

### Antisense Oligonucleotide (ASO) Targeting* APOC3: *Volanesorsen

The first ASO targeting *APOC3*, volanesorsen, blocks apo-CIII synthesis in the nucleus of hepatic cells by inhibiting *APOC3* mRNA. There are 2 major clinical trials with volanesorsen, the APPROACH trial [12••], its open label extension (OLE) trial [13•], and the COMPASS trial [14•].

#### The APPROACH Trial

This was the first phase 3 clinical trial with volanesorsen. It was a double-blind, randomized, placebo-controlled trial of 52 weeks examining whether the drug decreases TG levels compared to placebo in 66 patients with FCS. At 3 months, patients on volanesorsen 300 mg weekly subcutaneously (s.c.) had an 84% decrease in mean plasma apo-CIII while patients on placebo had a 6.1% increase (*P* < 0.001). Similarly, the mean TG levels in patients with volanesorsen decreased by 77% while in the placebo group there was an 18% increase (*P* < 0.001). About 77% patients on volanesorsen and 10% of the placebo group achieved TG levels < 750 mg/dl. While HDL cholesterol (HDL-C) was increased by 46%, LDL-C was also increased, as expected due to an increased lipolysis, by an impressive 136% and apolipoprotein B (apoB) by 20%. Hepatic fat fraction (HFF), a prespecified safety endpoint, was not significantly reduced after 1 year of therapy compared to baseline [15]. Regarding events of AP, an exploratory endpoint, there were 4 events in three placebo-treated patients and one event in one volanesorsen-treated patient. Regarding adverse events, injection-site reactions (ISR) occurred in 61% of the patients on volanesorsen and in none of the patients in the placebo group. Furthermore, none of the patients in the placebo group had platelet counts < 100,000/µl while 45,4% of patients in the volanesorsen group had such levels, with 2 cases of platelet counts < 25,000/µl. The platelet count comes back to normal after cessation of therapy and thrombocytopenia is most likely due to the chemistry of the 2'-*O*-methoxyethyl-modified ASOs [1•]. Biweekly monitoring of the platelet count is obligatory. This becomes more frequent if the platelet count decreases to < 100,000/µl and stopping rules are provided. Furthermore, quarterly controls of liver function tests and renal function is recommended.

Based on the results of the APPROACH trial volanesorsen was approved as treatment for patients with genetically confirmed FCS in the European Union and UK but approval was declined in the US based on its risk–benefit ratio.

#### The COMPASS Trial

The second phase 3 trial of volanesorsen was the COMPASS trial [14•]. This multi-center, randomized, double-blind, placebo-controlled trial of 26 weeks examined if treatment with volanesorsen, compared to placebo, reduced plasma TG in patients with multifactorial chylomicronemia syndrome (MCM or MCS), also known as multifactorial sHTG, in 114 subjects with fasting plasma TG > 500 mg/dl. Consistent with the findings of the APPROACH trial, after 3 months patients on volanesorsen 300 mg weekly s.c. had a 71.2% reduction in mean plasma TG from baseline compared with a 0.9% increase observed in the placebo group (*P* < 0.0001). Also similar to the APPROACH findings, chylomicron triglycerides, apo-B48, VLDL-cholesterol (VLDL-C) and non-HDL cholesterol (non-HDL-C) all showed significant reductions in the volanesorsen group. Also as reported before, HDL-C, apo-AI and LDL-C increased compared to baseline levels by 61.2%, 13.4% and 95.5%, respectively. ApoB increased by a non-significant 5.8%. The majority of the subjects had a strong polygenic basis of their sHTG, while 19% were heterozygous for a single loss-of-function variant in a canonical gene for FCS such as *LPL*, *APOC2* and *APOA5*. Interestingly, no differences between genotypes in response to treatment were observed. HFF, a prespecified exploratory endpoint, was significantly reduced [15]. Acute pancreatitis events, an exploratory endpoint, were only seen in the placebo group (5 events in 3 patients). Regarding adverse events (AEs) of interest, ISRs were recorded in 8% in the volanesorsen compared to none in the placebo group. In the volanesorsen group there was one case of thrombocytopenia with platelets < 50,000/µl and one case of serum sickness.

#### The APPROACH Open-label Extension (OLE) Trial

This phase 3 study aimed to evaluate the efficacy and safety of a long-term treatment with volanesorsen in 3 groups of patients with FCS [13•]. The first and second group included patients who had previously taken part in the APPROACH (*N* = 44) or in the COMPASS (*N* = 5) study, respectively, while the third group consisted of treatment-naive patients who had not participated in either study. Endpoints were change in fasting TG and other lipid parameters, as well as safety over 52 weeks. Volanesorsen reduced TG levels from index study baseline to months 3, 6, 12 and 24 by 48%, 55%, 50%, and 50%, respectively (APPROACH); by 65%, 43%, 42%, and 66%, respectively (COMPASS); and by 60%, 51%, 47%, and 46%, respectively in the treatment-naive population. These reductions were less than those reported in the primary efficacy time point of the parent trials (3 and 6 months, respectively), namely 77.0% in APPROACH and 71.2% in COMPASS and seem to decrease over time. This may be due to the dose-reductions or pauses of treatment being necessary during the trial because of thrombocytopenia. Overall, 65% of the patients had their dose reduced from weekly to every 2 weeks and 75% had a dose interruption.

There were no significant changes in the frequency or severity of patient-reported abdominal pain or in quality of life, results that are consistent with data from the initial APPROACH trial.

Regarding AP events, there was a reduction in both the number of patients experiencing on-treatment AP events compared with the previous 5 years (from 33 to 4) and the number of AP events (from 82 prior treatment to 4 on-treatment). However, without a placebo or non-treatment group, it cannot be evaluated whether treatment with volanesorsen reduces the risk of AP. AES were, as previously shown with volanesorsen, ISRs in 61.8% of the patients and platelet count decrease with 6.1% of the patients having a confirmed nadir platelet count post baseline between 25,000 and 50,000/µl.

#### Other Volanesorsen Trials

Volanesorsen has also been evaluated in other conditions associated with sHTG such as diabetes mellitus type 2 (*N* = 15, 15-week phase 2 study) [16] and familial partial lipodystrophy (FPL) (*N* = 40, 13-week phase 2/3 study), the BROADEN study [17]. Both trials showed significant decreases in TG levels from baseline of 69% and 88%, respectively. In the BROADEN trial volanesorsen significantly reduced HFF (secondary endpoint) [15]. Based on the BROADEN trial volanesorsen received approval in 2022 for the treatment of FPL in Brazil.

Recently the long-term (up to 51 months) efficacy and safety data of volanesorsen 285 mg s.c. every 2 weeks (a dose lower than that subsequently approved) were published [18]. The data are from 22 adults with genetically confirmed FCS treated in the UK through the Early Access to Medicines Scheme (EAMS) in patients that were treatment-naive (*n* = 12) or which had received volanesorsen in the APPROACH and APPROACH-OLE trials (*n* = 10). Reduction in TG levels were ~ 50% after 3 months of treatment and 10–38% after 21 months of treatment. A comparison of pancreatitis event rates found a 74% reduction from the 5-year period before (one event/2.8 years) and during (one event/11.0 years) volanesorsen treatment. Platelet decline frequency was observed similarly to the one reported in all phase 3 clinical trials.

### Antisense Oligonucleotide (ASO) Targeting *APOC3*: Olezarsen

Olezarsen is an advanced form of volanesorsen since this ASO is conjugated with N-acetylgalactosamine, an aminosugar with strong binding affinity for the asialoglycoprotein type 1 receptor, a mechanism that enhances targeted delivery to hepatocytes [19]. Till now there has only one phase 2 study with olezarsen been published.

In a phase 2 randomized, double-blind, placebo-controlled, dose-ranging study in 114 patients with moderate HTG (TG 200–500 mg/dl) participants received olezarsen in doses of 10 or 50 mg every 4 weeks, 15 mg every 2 weeks, or 10 mg every week or placebo s.c. for 6–12 months [20•]. Baseline median fasting TG was 262 mg/dl. TG were significantly reduced by 23% with 10 mg every 4 weeks, 56% with 15 mg every 2 weeks, 60% with 10 mg every week, and 60% with 50 mg every 4 weeks, while TG increased by 6% in the placebo group compared with an increase by 6% for the pooled placebo group. Significant reductions in apo-CIII, very low-density lipoprotein cholesterol (VLDL-C) and non-HDL-C were also seen. LDL-C levels changed minimally or increased with some doses, ranging from –1 to + 16%. The apoB changes ranged between + 2 to 16%, with no discernible dose–response pattern. There were no platelet count, liver, or renal function changes in any of the olezarsen groups. The most common AE were mild ISRs. A subsequent NMR-derived lipoprotein size and concentration analysis of this collective [21] showed that total HDL particle concentration increased by 15%, due primarily to a 32% increase in small HDL subspecies, a constellation that has been shown to be inversely related to mortality risk [22].

Five phase 3 studies are currently ongoing with olezarsen in patients with either FCS (NCT04568434) [23] or sHTG (> 500 mg/dl) (NCT05552326, NCT05079919) [24, 25] or with TG between 150–500 mg/dl (moderate HTG) and ASCVD or at increased risk of ASCVD (NCT05355402, NCT05610280) [26, 27].

### Small Interfering RNA targeting *APOC3*: ARO-APOC3

ARO-APOC3 is a GalNAc-conjugated small interfering RNA (siRNA) that targets *APOC3* mRNA. Unlike ASO, which act in the nucleus of the hepatocyte, siRNA acts mainly in the cytoplasm.

In a phase 1 study of subjects with HTG (TG ≥ 300 mg/dl) or MCM (TG ≥ 880 mg/dl) 50 mg ARO-APOC3 decreased TG levels up to 78% and 92%, respectively, compared to baseline 4 weeks after a single s.c. dose. HDL-C was increased by 71% and 136%, respectively. There were no reports of treatment-related serious or severe AE [28•].

In another phase 1 trial, 4 genetically confirmed FCS patients received 50 mg ARO-APOC3 and 26 patients with MCM (TG ≥ 880 mg/dl) received 10, 25, 50 or 100 mg ARO-APOC3 at baseline and after 4 weeks. Both groups showed similar maximum or median TG reductions of 91.3% and 89.8%, respectively. HDL-C was increased by 152.4% and 110.8%, respectively [29•]. It was reported that AEs were similar between groups, no further details were given.

There are 3 currently ongoing trials with ARO-APOC3, one phase 2b trial in patients with moderate HTG (NCT04998201) [30], one phase 2b trial in sHTG (> 500 mg/dl) (NCT04720534) [31] and a phase 3 trial in patients with FCS (NCT05089084) [32]. Their results will provide more information regarding the efficacy and safety profile of this very promising agent.

## Angiopoietin-like 3 (ANGPTL3) Inhibition

Angiopoietin-like 3 (ANGPTL3) protein is synthesized in the liver and modulates lipid metabolism mainly by inhibiting LPL and endothelial lipase (EL) [33]. Moreover, loss-of-function variants in *ANGPTL3* have been associated with decreased LDL-C, HDL-C and TG levels and with protection from ASCVD. Heterozygous carriers of LOF variants have a 41% lower risk of coronary artery disease than non-carriers [34]. These attributes made ANGPTL3 a very attractive new target for the treatments of hypercholesterolemia and sHTG.

### Small Interfering RNA Targeting *ANGPTL3*

ARO-ANG3 is a siRNA targeting *ANGPTL3*. In a 16-week study of healthy volunteers (NCT03747224) [35], treatment with ARO-ANG3 (100–300 mg s.c.) lowered plasma TG by 61–65%, LDL-C by 45–54%, and HDL-C by 14–37% 12 weeks after the second dose (dosing was performed at days 1 and 29) without apparent dose response [36]. In another 16-week phase 1 study of patients with hypercholesterolemia there was a 25–43% decrease in TG levels from baseline in patients with heterozygous familial hypercholesterolemia (FH) (open label study, dose range 100–300 mg) with no apparent dose response and in non-FH patients (dose 200 mg) a 29% decrease (vs. a 14% decrease in the placebo group) 12 weeks after the second dose (dosing was performed at day 1 and 29) [37••].

There are currently 2 ongoing trials with ARO-ANG3, one phase 2b trial in patients with mixed dyslipidemia (NCT04832971) [38] and one phase 2 trial in patients with homozygous FH (HoFH) (NCT05217667) [39]. There are also plans to initiate a trial in patients with sHTG in late 2023 (Prof. Gerald Watts, personal communication).

A summary of the emerging treatments for sHTG can be found in Table 1.

**Table 1:** Emerging therapies for severe hypertriglyceridemia (sHTG)

Agent	Dose	Mechanism of action	Main indication	Comments
Class: Apo-CIII inhibitors				
Volanesorsen	300 mg s.c. once a week	ASO inhibiting *APOC3*	TG reduction	↓ TG by 50–70%; approved in EU and UK for FCS but not in North America
Olezarsen	Unspecified (range 10–50 mg) s.c. every 4 weeks	ASO inhibiting *APOC3*	TG reduction	↓ TG by 50–70%; GalNAc-linked ASO; Phase 3 trials ongoing
AROAPOC3	50 mg s.c. every 12 weeks	siRNA inhibiting *APOC3*	TG reduction	↓ TG up to 90% in phase 1 trials; GalNAc-linked siRNA; Phase 3 trials ongoing
Class: ANGPTL3 inhibitors				
AROANG3	Unspecified (range 100–300 mg), s.c. every 12 weeks	ASO inhibiting *ANGPTL3*	LDL-C and TG reduction	TG ↓ up to 65%, LDL-C ↓ up to 55% in phase 1 trials; GalNAc-linked siRNA; Phase 2 trials ongoing
LY3475766	Unspecified, s.c.	mAb targeting ANGPTL3/8 complex	LDL-C and TG reduction	↓ TG up to 70%, LDL-C ↓ up to 37% in phase 1 trials; further development unclear

*ASO* antisense oligonucleotides; *siRNA* small interfering RNAs; *mAb* monoclonal antibodies

### ANGPTL3/8 Inhibition: LY3475766, Monoclonal Antibody Against ANGPTL3/8

ANGPTL8 is a cofactor of ANGPTL3 efficacy [40]. ANGPTL8 is secreted in response to feeding and forms a complex with ANGPTL3. An ANGPTL8 LOF variant has been associated with lower TG and LDL-C levels and, in contrast to ANGPTL3 LOF, with increased HDL-C levels [41]. However, because this variant is very rare (frequency 0.01%) the study was not sufficiently powered to assess cardiovascular protection. The ANGPTL3/8 complex inhibits LPL 100-fold more potently and circulates at much lower levels than ANGPTL3 alone [40], making it an interesting potential therapeutic target for the treatment of HTG and hypercholesterolemia.

In a phase 1, double-blind, placebo-controlled, single ascending dose study LY3475766, a monoclonal antibody against the ANGPTL3/8 complex was investigated in 48 individuals with mixed hyperlipidemia (TG ≥ 135 mg/dl and LDL-C ≥ 70 mg/dl) over 28 days (NCT04052594) [42]. The results of this study were presented as an oral presentation by the first author Daniel Gaudet at the 90^th^ European Atherosclerosis Society (EAS) Congress in Milan Italy, 22–25 May 2022. There were 5 cohorts of LY3475766 receiving each 10 mg and 300 mg i.v. or 100 mg, 300 mg and 600 mg s.c. TG levels decreased up to 70%, LDL-C up to 37%, apoB up to 31%. HDL-C increased up to 26%. Decreases were dose-dependent and maximal with the 600 mg s.c. dose.

There were no serious AEs and no treatment-emergent AEs (TEAEs). There were 4 cases of mild ISR in the active-treated subjects and one in the placebo group.

Further studies are needed to establish the role LY3475766 in the treatment of sHTG.

## Recently Suspended Drug Therapies for sHTG

### Monoclonal Antibody Against Angiopoietin-like Protein 3 (ANGPTL3): Evinacumab

Evinacumab, a monoclonal antibody binding ANGPTL3 is already approved in the EU, UK and the US for the treatment of HoFH. It is given i.v. once a month at a dose of 15 mg/kg. A phase 2, placebo-controlled, randomized trial with evinacumab examined its potential for the treatment of sHTG [43]. The study examined 3 groups of patients, all with sHTG and a history of AP, with either FCS, with MCM due to heterozygous LOF LPL pathway mutations or those with MCM without such mutations. There was no decrease in TG in the FCS group (–27.7% vs. –22.9% in the placebo group). In the group of MCM with heterozygous LOF mutations there was a significant TG decrease of 64.8% vs. a 9.4% increase in the placebo group, and an even greater TG decrease was seen in the third group of MCM patients with no such mutations (–81.7% vs. an 80.9% increase in the placebo group). The results show that for evinacumab to be able to decrease TG, some LPL activity needs to be present, meaning that it can be used as a treatment for the vast majority of the sHTG cases.

However, a phase 2 trial with evinacumab in patients with sHTG (with a history of acute pancreatitis) to prevent acute pancreatitis was stopped in 2023 (sponsor's decision, Regeneron) due to poor recruitment (personal communication) (NCT04863014) [44]. Probably the i.v. mode of administration of evinacumab makes it a not so attractive option for treating sHTG. There will be no further development of evinacumab for this indication (sponsor's decision, personal communication).

### ASO Targeting *ANGPTL3*: Vupanorsen

Vupanorsen is a GalNAc-conjugated ASO targeting ANGPTL3 mRNA and thus inhibiting ANGPTL3 protein synthesis. There were promising initial results with vupanorsen. In a phase 1 trial in 32 healthy volunteers, a 33.2–63.1% reduction in TG levels was achieved as well as a 1.3–32.9% decrease in LDL-C and a 3.4–25.7% decrease in apoB, depending on the dose administered (10–60 mg per week for 6 weeks s.c.) [45]. Moreover, a 6-month, double-blind, placebo-controlled, dose-ranging phase 2 trial in 105 patients with fasting TG > 150 mg/dl (median baseline TG were 252 mg/dl), type 2 diabetes, and hepatic steatosis treated with 20–80 mg of vupanorsen showed significant reductions in TG concentrations of up to 53% compared with a 16% reduction with placebo [46]. Non-HDL-C was also decreased by a maximum of 18% as well as HDL-C by 24%. LDL-C levels remained unchanged. The highest dose (80 mg q4w) was associated with significant increases in alanine aminotransferase (ALT) and aspartate aminotransferase (AST) as well of the HFF. However, a subsequent dose-finding phase 2b study in 286 patients with moderate HTG on statins produced some surprising results that led to the discontinuation of further development of the drug [47]. While there was a rather modest TG (up to 56.8%) and non-HDL-C (up to 27.7%) reduction, there was a massive dose-dependent increase in hepatic fat (up to 76%). Moreover, higher doses were associated with > 3 × elevations in the liver enzymes ALT and AST. Therefore, there was no support by the sponsor to continue the clinical development program of the drug, which was discontinued in January 2022.

Of note, this liver-associated toxicity has neither been observed with evinacumab nor in patients with ANGPTL3 LOF mutations and ANGPTL3 inhibition in mice with an ASO led to a decrease in liver triglyceride content [45, 48]. In addition, circulating ANGPTL3 levels are not associated with non-alcoholic fatty liver disease [49]. Therefore, it will be interesting to see if the siRNA ARO-ANG3 is also associated with similar increases in HFF and liver enzymes as vupanorsen. It is still unclear if the significant hepatic side effects observed with vupanorsen were an off-target effect of intracellular ANGPTL3 inhibition or an effect specifically associated with this drug.

A summary of the recently abandoned treatments for sHTG can be found in Table 2.

**Table 2:** Recently discontinued therapies for severe hypertriglyceridemia (sHTG)

Agent	Mechanism of action	Year and reason development was discontinued
Evinacumab	Monoclonal antibody targeting ANGPTL3	2023, poor recruitment in pivotal study
Vupanorsen	ASO targeting *ANGPTL3*	2022, increase in hepatic fat

*ASO* antisense oligonucleotides

## Conclusions

In recent years the development of novel pharmacological agents for the treatment of sHTG has been an area of great interest for clinicians, researchers and for the pharmaceutical industry. There is up to now a disconcerting lack of effective and safe therapies for this indication to help decrease the substantially increased risk of pancreatitis associated with this condition. In our opinion the two most promising therapeutic targets being currently studied are apo-CIII and ANGPTL3. Blocking *APOC3* decreases TG independently from the LPL lipolytic pathway while anti-ANGPTL3 therapies such as evinacumab seem to require some LPL-associated lipolytic pathway activity. Therefore, while the former approach could be used in all forms of sHTG, the latter would be ineffective in the rare FCS patients.

Volanesorsen, a second-generation ASO targeting apo-CIII, is an effective treatment for sHTG, albeit approved only for patients with FCS. Furthermore, widespread use of volanesorsen has been limited by off-target side effects such as ISRs and mainly drug-induced thrombocytopenia, which can be quite pronounced in some patients and the mechanism of which has not been definitely clarified. Moreover, the burden of frequent laboratory controls for patients and treating physicians and the overall treatment costs should not be underestimated.

There are, however, very promising new treatments for sHTG also via apo-CIII inhibition in various phases of development. Olezarsen, a third generation ASO also targeting apo-CIII, conjugated with the ligand N-acetylgalactosamine (GalNAc) decreases TG by ~ 60% in patients with moderate hypertriglyceridemia, with studies on patients with sHTG approaching completion. Since GalNAc-associated ASOs have a high-affinity for the hepatocyte-specific ASGL receptor, olezarsen will probably require much smaller doses (< 10%) than volanesorsen, has a longer duration of action and fewer side effects. It may be that olezarsen will be the new, improved and safer volanesorsen. ARO-APOC3 also seems to be very efficacious and safe with phase 2 and 3 studies on patients with sHTG approaching completion.

Regarding ANGPTL3 inhibition to treat sHTG, ARO-ANG3 is the only candidate under development after the cancellation of the vupanorsen and evinacumab programs.

Studies with ARO-ANG3 will answer the interesting question whether intracellular targeting of ANGPTL3 with this siRNA has the same hepatic side effects that led to the demise of vupanorsen.

Other therapeutic modalities such as liver-targeted genome editing either as standard nuclease genome editing or base editing, have been used to inactivate *PCSK9* and *ANGPTL3* in non-human primates for the treatment of hypercholesterolemia and HTG [50] but whether these methods will be successfully applied to humans remains to be investigated.

The totality of evidence suggests that ANGPTL3 inhibition seems to be a better target for the treatment of hypercholesterolemia than sHTG, while apo-CIII seems to be much more promising for the treatment of sHTG and has at best no or negative effects on LDL-C levels.

Olezarsen, ARO-APOC3 and especially ARO-ANG3 have also a mechanistic potential to reduce the risk of ASCVD in patients with HTG but such studies need to be performed separately with the respective endpoints; they are already underway with olezarsen.

While all these new promising drugs are effective in decreasing TG, the holy grail of sHTG treatment remains the reduction in the incidence of AP. Whichever drug provides the most robust evidence in this regard, also considering treatment costs, will be the ultimate winner in the race for the best drug to treat sHTG.
